# Dopamine Agonist Increases Risk Taking but Blunts Reward-Related Brain Activity

**DOI:** 10.1371/journal.pone.0002479

**Published:** 2008-06-25

**Authors:** Jordi Riba, Ulrike M. Krämer, Marcus Heldmann, Sylvia Richter, Thomas F. Münte

**Affiliations:** 1 Department of Neuropsychology, University of Magdeburg, Magdeburg, Germany; 2 Departament de Farmacologia i Terapéutica, Centre d'Investigació del Medicament, Institut de Recerca, Servei de Farmacologia Clínica, Hospital de la Santa Creu i Sant Pau, Universitat Autònoma de Barcelona, Barcelona, Spain; 3 Department of Neurology II, University of Magdeburg, Magdeburg, Germany; 4 Department of Neurochemistry and Molecular Biology, Leibniz Institute for Neurobiology, Magdeburg, Germany; 5 Center for Behavioral Brain Sciences, Magdeburg, Germany; James Cook University, Australia

## Abstract

The use of D2/D3 dopaminergic agonists in Parkinson's disease (PD) may lead to pathological gambling. In a placebo-controlled double-blind study in healthy volunteers, we observed riskier choices in a lottery task after administration of the D3 receptor-preferring agonist pramipexole thus mimicking risk-taking behavior in PD. Moreover, we demonstrate decreased activation in the rostral basal ganglia and midbrain, key structures of the reward system, following unexpected high gains and therefore propose that pathological gambling in PD results from the need to seek higher rewards to overcome the blunted response in this system.

## Introduction

The administration of dopaminergic drugs with D2/D3 agonist activity to Parkinson's patients, targeting the dopaminergic deficit in the nigrostriatal pathway in this condition, has recently been reported to lead to impulse control disorders, in particular pathological gambling, suggesting a breakdown of reward-hierarchies in such patients [1]–[3]. In the survey conducted by Weintraub and colleagues [3], about 7 percent of Parkinson's patients treated with the popular D2/D3 receptor-preferring agonist pramipexole developed pathological gambling, while none of the patients receiving standard treatment with dopamine precursor L-Dopa showed this condition. This suggests that the processing of rewarding stimuli might be altered under the influence of D2/D3 agonists.

The underlying neural mechanism by which these dopaminergic agents modify the processing of rewarding stimuli and lead to compulsive gambling remains poorly understood. A recent fMRI study employing a gambling task found a decreased sensitivity of the reward system in pathological gamblers [4]. Activation of the ventral striatum following wins was found to be decreased compared to healthy control subjects and correlated negatively with gambling severity [4]. The intensity of the fMRI BOLD signal in the nucleus accumbens (NAcc) of the ventral striatum has been found to be directly related with dopamine release in this structure [5]. In the light of these findings we hypothesized that dopaminergic D2/D3 agonists may impair the responsiveness of brain reward systems. Consequently, we expected that neural activation associated with monetary reward in a gambling task should be reduced under treatment with pramipexole.

**Figure 1:**
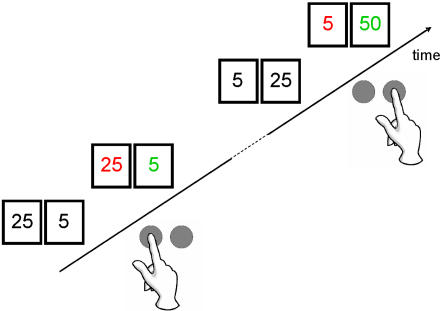
Experimental paradigm. Each trial of the lottery task comprised the presentation of two numbers, 25 and 5. Participants had to choose one of two numbers by pressing a corresponding button (left button for left number). One second after the participant's selection, the numbers changed color. Green indicated a win, red a loss. Thus, in the first (lower) example the subject incurred a loss of 25 Euro cent (while he could have won 5 Euro cent). The second (upper) example shows an infrequent “boost” trial in which gains were doubled. See the Materials and Methods section for further details.

In order to test this hypothesis, we performed a slow event-related fMRI study in a group of 15 healthy young male volunteers using a 3 Tesla scanner. A double-blind, placebo-controlled cross-over design was used with placebo given in one session and 0.5 mg of pramipexole given 2 hours prior to the scan in the other. A lottery task was used in which participants could either win or lose 25 or 5 Euro cents in each trial (see **Fig. 1**). In addition, in a small proportion of the trials, wins were doubled. The rationale for the inclusion of such “boost” trials was the finding in animal research that unexpected wins lead to an increase in phasic activity of midbrain dopaminergic neurons projecting (among other structures) to the NAcc [6]. We thus predicted that reward-related activation of the striatum would be maximal in the boost trials.

## Results

**Figure 2:**
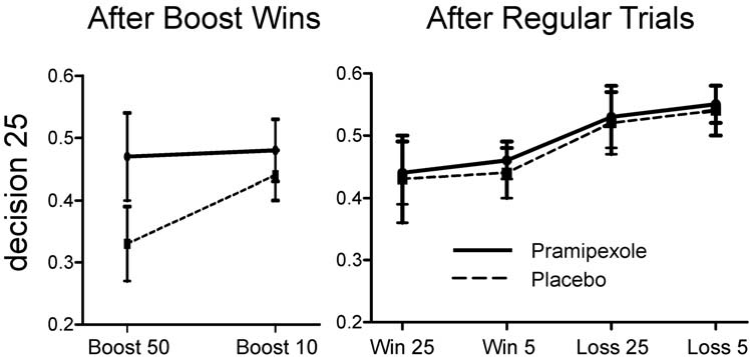
Behavioral results. The relationship between outcome in the previous trial and the probability of making a risky choice (choosing 25) is shown. Pramipexole significantly increased the probability of making a risky choice following an unexpected “boost+50” trial (left). The error bars denote ±1 standard error of mean.

The behavioral data (**Fig. 2**) showed a characteristic relationship between *previous* outcomes and *current* choices. The analysis of the standard outcomes by means of a three-way ANOVA with treatment (placebo vs. pramipexole), type of previous outcome (gain vs. loss) and magnitude of outcome (25 or 5) showed a significant effect of type of previous outcome [F(1,12) = 12.4; p<0.01], with losses in the previous trial leading to riskier choices (i.e., higher probability of selecting 25) than gains. Although, the overall number of times that subjects made a risky choice (25 cents) did not differ between treatments: 47% after placebo and 49% after pramipexole (t(12) = −1.0, p = 0.33); the analysis of the infrequent boost trials by means of a two-way ANOVA with treatment and type of boost trial (“+50” vs. “+10”) as factors showed a main effect of treatment [F(1,12) = 5.0, p<0.05]. Pairwise comparisons by means of t-tests showed a significant effect for the “+50” trials only; t(12) = −2.28, p<0.05. Thus, pramipexole abolished the tendency to be conservative after a boosted win of 50 that was present under placebo. This behavioral pattern is consistent with clinical observations of an increase of risk behavior in Parkinson's patients treated with dopaminergic agonists.

**Figure 3:**
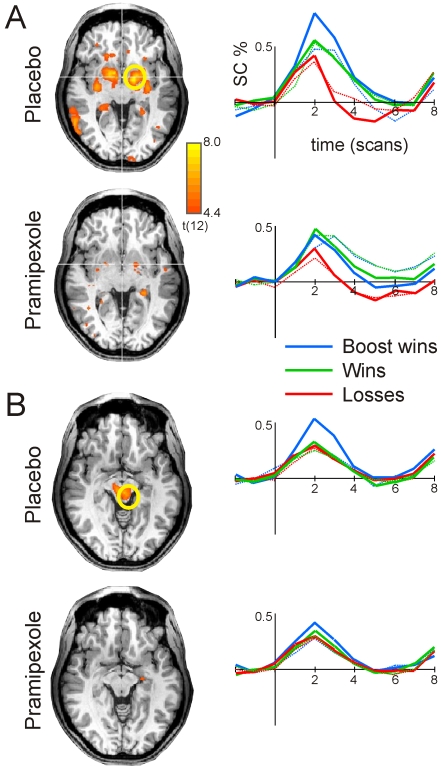
(a) fMRI results (rostral basal ganglia). Axial slices at z = −3 showing voxels of greater activation for win (normal gain+boost) than loss trials in the ventral striatum after placebo and pramipexole. The time course of the BOLD response is shown for a ROI defined around voxel x = −15,y = −3,z = −3 in Talairach coordinates. Results are shown at p<0.001 uncorrected (n = 13). (b) fMRI results (midbrain). Axial slices at z = −8 showing the results of a conjunction analysis conducted to highlight those voxels where activation following unexpected high gains (boost+50 trials) was greater than the activation following normal gain trials and unexpected boost+10 trials after placebo and pramipexole. The time course of the BOLD response is shown for a ROI defined around voxel x = −4,y = −23,z = −8 in Talairach coordinates. Results are shown at p<0.0002 uncorrected (n = 13). In both panels (a and b), the color code is as follows: win trials (green; solid: 25 cent wins, dotted: 5 cent wins); loss trials (red; solid: 5 cent loss, dotted: 25 cent loss); boost trials (blue; solid: 50 cent wins, dotted: 10 cent wins).

Functional MRI results showed that monetary gains (standard gain plus boost trials vs. losses) in the placebo condition robustly increased activity in the rostral basal ganglia, in an area encompasing the globus pallidus and parts of the ventral striatum (**Fig. 3a**). At the same threshold, this activation was greatly diminished under pramipexole. To assess the response pattern in more detail, the time-course of the blood oxygen level dependent (BOLD) response in rostral basal ganglia was examined by defining a region of interest (ROI) around the peak of activation (*x*,*y*,*z*: −15,−3,−3) in the globus pallidus. In the placebo condition, win trials (green; solid: 25 cent wins, dotted: 5 cent wins) showed an increase in BOLD relative to loss trials (red). In particular the 50 cent boosted wins (blue thick line) gave rise to a pronounced increase of activity. By contrast, this increased response to unexpected high gains was absent in the pramipexole condition. Percentage bold increase to the boosted +50 condition under pramipexole was lower (0.58%) than after placebo (0.80%), t(12) = −2.44, p<0.05, potentially reflecting decreased phasic dopamine release [5].

Additionally, under placebo, unexpected high gains (boost +50) were associated with higher midbrain activity relative to normal gains (+5, +25) and +10 boost trials (see **Fig. 3b**), in line with data from animal studies (e.g., [6]). Interestingly, this increase in neural activity was decreased under pramipexole. In addition to the voxel-based analysis, the BOLD response for a ROI defined around the peak of activation (*x*,*y*,*z*:−4,−23,−8) was also assessed. Similarly to the results found in the rostral basal ganglia, the BOLD increase to the boost +50 trials (dark blue line) was lower under pramipexole (0.44%) than in the placebo condition (0.58%), t(12) = −2.24, p<0.05.

**Figure 4:**
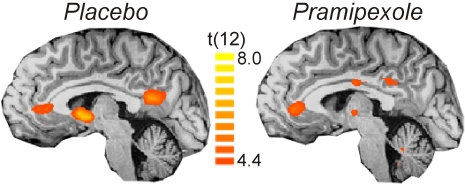
fMRI results (cingulate gyrus). Sagittal slices at x = −4 showing voxels of greater activation for win (normal gain+boost) than loss trials in the rostral anterior and posterior cingulate gyrus. Results are shown at p<0.001 uncorrected (n = 13).

While our main focus was on the responses in the ventral striatum and the midbrain, we also found significant activations in the contrast (standard gain plus boost trials vs. losses) in the anterior cingulate gyrus (x,y,z coordinates: −6, 43, 7) and the posterior cingulate gyrus (x,y,z coordinates: −6, −52, 16, see figure 4) in both, placebo and pramipexole conditions (see [7] for similar activations).

## Discussion

Results in the present study suggest that under pramipexole subjects make riskier choices following unexpected high wins than when under placebo. Furthermore, unexpected high wins appear to loose their highly rewarding quality under pramipexole treatment, as indicated by the decreased activation of the rostral basal ganglia This area, especially the ventral striatum, has been hypothesized to subserve the integration of reward signals in particular with regard to the planning of future behavior [8]. Additionally, decreased activation was also observed in the midbrain. The region of blunted activation is at the height of and appears to include the ventral tegmental area and the substantia nigra, although the peak coordinates do not coincide with these regions. A diminished response to unexpected high wins suggests that integration of reward signals is deficient in the drug condition. The participants' strategy of making riskier choices could be interpreted as a behavioral attempt to compensate for this decreased activation. This might be the basis for the high rate of impulse control disorders in Parkinson's patients receiving dopaminergic agonists.

The pharmacologically induced hypoactivation of reward circuits was analogous to that observed in pathological gamblers not suffering from neurological disorders [4]. Compulsive gambling in these patients has been interpreted as an attempt to compensate for an insufficient dopaminergic activation [4]. In fact, current theories trying to explain other impulse controls disorders such as compulsive drug taking postulate that in the transition from recreational drug use to addiction a decrease in the functionality of reward systems occurs prior to the instatement of compulsive drug-seeking behavior [9]–[11].

Various impulse control disorders characterized by exaggerated activities with high hedonic value have been described for Parkinson patients after treatment with dopaminergic agents [3], [12]. These behaviors could constitute maladaptive attempts to recover the normal operating level of a hypoactive reward system. An explanation for these undesired side effects may be found in the interaction of pramipexole with the striatal D2 and D3 receptors. D2 autoreceptor agonism in the striatum has been postulated as the explanatory mechanism for the observed blunted BOLD signal to reward predicting stimuli in the NAcc following oral amphetamine in healthy volunteers [13]. These autoreceptors are located on presynaptic neurons and serve to regulate neurotransmitter release by activating negative feedback loops when neurotransmitter concentrations in the synaptic cleft reach certain levels. Drugs activating presynaptic autoreceptors are typically able to decrease neurotransmitter release below physiological levels. Analogously, excessive chronic dopamine release in the NAcc has been related to a reduction in the BOLD signal during reward anticipation in schizophrenics [14]. Regarding D3 receptors, these are located in the ventral striatum, but also in the midbrain, where they act as autoreceptors [15]. In this respect, D3 autoreceptor activation has been found to inhibit the reward-related phasic firing of dopaminergic neurons [15]. D3 receptors have been shown to play a relevant role in animal models of drug dependence, with D3 antagonists consistently inhibiting drug-administration and drug-seeking behavior in rodents [16]–[18]. A D3 receptor mediated inhibition of midbrain nuclei would be consistent with the reduced midbrain activation observed under pramipexole in the present study.

In summary, the present findings indicate that the dopamine D2/D3 receptor agonist pramipexole is capable of blocking reward-related activations in the rostral basal ganglia and midbrain and may lead to a behavioral disinhibition characterized by increases in risky choices in a gambling task. Drug-induced hyporesponsiveness of reward circuits may underlie the impulse control disorders observed in Parkinson patients treated with dopaminergic agonists.

## Materials and Methods

All procedures had been approved by the ethical committee of the University of Magdeburg.

### Participants

Fifteen healthy male volunteers participated in the study, which comprised two experimental sessions. None of the participants had a history of neurological or psychiatric disorders and their ages ranged from 20 to 31 years (mean = 24.4 years). All subjects gave their written informed consent and were paid for participation.

### Drugs and study design

The study was conducted according to a double-blind randomized crossover design. All volunteers received the two study medications, i.e., pramipexole and placebo, each subject acting thus as his own control. Volunteers participated in two different experimental sessions, separated at least by one week. On each experimental session participants received 20 mg domperidone in a non-blind fashion in order to antagonize any potential nausea induced by pramipexole. One hour later they received either placebo (lactose) or 0.5 pramipexole hydrochloride in a double blind fashion according to a randomization table. Half of the subjects received placebo in the first experimental session and pramipexole in the second. The other half received pramipexole in the first session and placebo in the second. The fMRI procedures were started two hours after pramipexole/placebo administration.

### Task and stimuli

The experimental paradigm consisted in a lottery task in which participants had to bet on one of two different numbers, 25 or 5, in order to increase a starting amount of 500 euro cents. Participants were instructed to choose one of the two numbers by button press. One second after the selection, the numbers changed color. Green indicated a win (i.e., +5 or +25), red a loss (i.e., −5 or −25). This feedback was shown for 2 s. The experimental session comprised two runs of 94 trials each. In 80 of the 94 trials, the feedback indicated a standard win or a standard loss. Additionally, in fourteen of the 94 trials, the so-called “boost trials”, wins were unexpectedly doubled and participants won 10 cents after choosing 5 (a green “10” was shown as feedback on the screen) and 50 cents after choosing 25 (a green “50” was shown as feedback on the screen). Thus, the six possible outcomes were: win 5, win 25, lose 5, lose 25, win 10 (“boost+10”) and win 50 (“boost+50”). No boost losses were programmed in the experimental paradigm. Participants were told that they had to adjust their choices in order to maximize their wins. In actual fact, the task was programmed to yield wins in 50% of the trials and losses in the other 50%. Participants were informed on the money they had won at three different times during each block. A slow event-related design was used with a constant interstimulus interval of 12 s, during which a fixation cross was presented in the center of the screen.

### Data acquisition and analysis

#### Acquisition

Data were acquired in a 3-Tesla Siemens Magnetom Trio Scanner. First, structural images of the brain were obtained by means of a T1-weighted MPRAGE sequence: 256×256 matrix; field of view (FOV) = 256 mm; 192 1-mm sagittal slices. Subsequently, functional images were obtained in two runs implementing an echo-planar-imaging sequence. The pulse-sequence parameters were as follows: time to repeat (TR) = 2000 ms; time to echo (TE) = 30 ms; FOV = 224 mm; flip angle (FA) = 80°; matrix = 64×64; slice thickness = 4 mm. Thirty-two transversal slices (3.5×3.5×4 mm voxel) were obtained parallel to the anterior commissure-posterior commissure (AC-PC).

#### Analysis

Usable data were finally obtained from 13 volunteers. One volunteer withdrew from the study after complaining of visual distortions which disappeared when he was removed from the scanner. Another participant invariably chose “25” in 100% of the trials throughout the two experimental sessions, thus failing to show any attempt to modulate his behavior based on prior outcomes.

#### *fMRI*

Data analysis included preprocessing (3D motion correction, slice scan time correction, high-pass temporal filtering and spatial smoothing with a 4 mm Gaussian filter, full-width-half-maximum), co-registration and normalization to Talairach stereotaxic space using Brain Voyager QX. Random-effects analysis was performed for the functional data (% of BOLD signal change) including the following predictors: “win+25”, “win+5”, “loss-5”, “loss-25” and “boost+10” and “boost+50” for the placebo and pramipexole conditions. Predictors were convolved with a two-gamma hemodynamic response function with the following parameter values: onset = 0, time to peak = 5 s, time to undershoot peak = 15 s.

Results were considered statistically significant when p values were lower than 0.001 uncorrected for multiple comparisons.

The time course of the BOLD response was assessed by defining regions of interest ROIs around the voxel of maximum statistical difference. The ROIs defined extended 5 voxels in the x,y,z directions. For direct statistical comparisons of ROI activation in a given condition between treatments, the maximum percentage of increase was obtained for each participant and these values compared by means of pairwise t-tests. Results were considered statistically significant when p values were lower than 0.05.

#### Behavior

The probability of making “risky” choices, i.e., choosing 25, was measured in relation to the outcome on the previous trials. These values were analyzed by means of repeated measures ANOVAs. Factors included treatment (placebo, pramipexole), type of previous outcome (gain, loss) and magnitude of outcome (25, 5, “boost+10” and “boost+50”). Results were considered statistically significant when p values were lower than 0.05.

## References

[1] Dodd ML, Klos KJ, Bower JH, Geda YE, Josephs KA (2005). Pathological gambling caused by drugs used to treat Parkinson disease.. Arch Neurol.

[2] Driver-Dunckley E, Samanta J, Stacy M (2003). Pathological gambling associated with dopamine agonist therapy in Parkinson's disease.. Neurology.

[3] Weintraub D, Siderow AD, Potenza MN, Goveas J, Morales KH (2006). Association of dopamine agonist use with impulse control disorders in Parkinson disease.. Arch Neurol.

[4] Reuter J, Raedler T, Rose M, Hand I, Glascher J (2005). Pathological gambling is linked to reduced activation of the mesolimbic reward system.. Nat Neurosci.

[5] Knutson B, Gibbs SE (2007). Linking nucleus accumbens dopamine and blood oxygenation.. Psychopharmacol.

[6] Hollerman JR, Schultz W (1998). Dopamine neurons report an error in the temporal prediction of reward during learning.. Nat Neurosci.

[7] Nieuwenhuis S, Slagter HA, von Geusau NJ, Heslenfeld DJ, Holroyd CB (2005). Knowing good from bad: differential activation of human cortical areas by positive and negative outcomes.. Eur J Neurosci.

[8] Delgado MR (2007). Reward-Related Responses in the Human Striatum.. Ann N Y Acad Sci.

[9] Ahmed SH, Kenny PJ, Koob GF, Markou A (2002). Neurobiological evidence for hedonic allostasis associated with escalating cocaine use.. Nat Neurosci.

[10] Kenny PJ (2007). Brain reward systems and compulsive drug use.. Trends Pharmacol Sci.

[11] Koob GF, Le Moal M (2005). Plasticity of reward neurocircuitry and the ‘dark side’ of drug addiction.. Nat Neurosci.

[12] Voon V, Hassan K, Zurowski M, de Souza M, Thomsen T (2006). Prevalence of repetitive and reward-seeking behaviors in Parkinson disease.. Neurology.

[13] Knutson B, Bjork JM, Fong GW, Hommer D, Mattay VS (2004). Amphetamine modulates human incentive processing.. Neuron.

[14] Juckel G, Schlagenhauf F, Koslowski M, Wüstenberg T, Villringer A (2006). Dysfunction of ventral striatal reward prediction in schizophrenia.. Neuroimage.

[15] Sokoloff P, Diaz J, Le Foll B, Guillin O, Leriche L (2006). The dopamine D3 receptor: a therapeutic target for the treatment of neuropsychiatric disorders.. CNS Neurol Disord Drug Targets.

[16] Ashby CR, Paul M, Gardner EL, Heidbreder CA, Hagan JJ (2003). Acute administration of the selective D3 receptor antagonist SB-277011A blocks the acquisition and expression of the conditioned place preference response to heroin in male rats.. Synapse.

[17] Di Ciano P, Underwood RJ, Hagan JJ, Everitt BJ (2003). Attenuation of cue-controlled cocaine-seeking by a selective D3 dopamine receptor antagonist SB-277011-A.. Neuropsychopharmacology.

[18] Thanos PK, Katana JM, Ashby CR, Michaelides M, Gardner EL (2005). The selective dopamine D3 receptor antagonist SB-277011-A attenuates ethanol consumption in ethanol preferring (P) and non-preferring (NP) rats.. Pharmacol Biochem Behav.

